# The dynamics of nocturnal sap flow components of a typical revegetation shrub species on the semiarid Loess Plateau, China

**DOI:** 10.3389/fpls.2024.1370362

**Published:** 2024-03-21

**Authors:** Weiwei Fang, Jianbo Liu, Nan Lu, Ruiping Li

**Affiliations:** ^1^ Key Research Institute of Yellow Civilization and Sustainable Development and Collaborative Innovation Center of Henan Province, Henan University, Kaifeng, China; ^2^ Tianjin Key Laboratory of Water Resources and Environment, Tianjin Normal University, Tianjin, China; ^3^ State Key Laboratory of Urban and Regional Ecology, Research Center for Eco-Environmental Sciences, Chinese Academy of Sciences, Beijing, China; ^4^ University of Chinese Academy of Sciences, Beijing, China

**Keywords:** nighttime transpiration, nighttime recharge, soil water content, stem water potential, Loess Plateau

## Abstract

**Introduction:**

The components of nighttime sap flux (En), which include transpiration (Qn) and stem water recharge (Rn), play important roles in water balance and drought adaptation in plant communities in water-limited regions. However, the quantitative and controlling factors of En components are unclear.

**Methods:**

This study used the heat balance method to measure sap flow density in *Vitex negundo* on the Loess Plateau for a normal precipitation year (2021) and a wetter year (2022).

**Results:**

The results showed that the mean values were 1.04 and 2.34 g h^-1^ cm^-2^ for Qn, 0.19 and 0.45 g h^-1^ cm^-2^ for Rn in 2021 and 2022, respectively, and both variables were greater in the wetter year. The mean contributions of Qn to En were 79.76% and 83.91% in 2021 and 2022, respectively, indicating that the En was mostly used for Qn. Although the vapor pressure deficit (VPD), air temperature (Ta) and soil water content (SWC) were significantly correlated with Qn and Rn on an hourly time scale, they explained a small fraction of the variance in Qn on a daily time scale. The main driving factor was SWC between 40-200 cm on a monthly time scale for the Qn and Rn variations. Rn was little affected by meteorological and SWC factors on a daily scale. During the diurnal course, Qn and Rn initially both declined after sundown because of decreasing VPD and Ta, and Qn was significantly greater than Rn, whereas the two variables increased when VPD was nearly zero and Ta decreased, and Rn was greater than Qn.

**Discussion:**

These results provided a new understanding of ecophysiological responses and adaptation of *V. negundo* plantations to increasing drought severity and duration under climate changes.

## 1 Introduction

As an important component of plant water physiology, nighttime sap flow (En) has been studied in various ecosystems and the contribution of En to total daily sap flow is approximately 1-28% across a range of habitats (Zeppel et al., 2010; Siddiq and Cao, 2018; Hayat et al., 2021; Yu et al., 2023). This value is naturally greater for water-limited regions because the higher En for stem water recharge to adapt the water scarcity (Snyder et al., 2003). En has been also considered to be potentially significant for water and surface hydrology (Zeppel et al., 2008), and plant and ecosystem carbon relationships (Wu et al., 2020). If potential changes in climate create warmer, drier conditions at night (increased vapor pressure deficit (VPD)), this would increase the percentage of En, especially under water-limited regions (Snyder et al., 2003). Therefore, nighttime water consumption patterns and plant physiological processes are important for understanding plant-species functioning in the future.

The response of En or nighttime stomatal conductance to VPD has been found to decline (Cavender-Bares et al., 2007), remain unchanged (Barbour et al., 2005; Wang et al., 2012), or increase (Zeppel et al., 2012; Wu et al., 2020) with increasing VPD. The influence of soil moisture on En can be as low as nearly negligible (Zeppel et al., 2010), positive (Chen et al., 2020), or irrelevant (Fang et al., 2018). The En process involves nighttime transpiration (Qn) and stem water storage/refilling (Rn). The main reason may be that the environmental factors have different effects on Qn and Rn, leading to the different effect of environmental factors on En. Therefore, by analyzing the influencing factors for each component of En, the variation of En could be better clarified.

These two components of En have different effects on plant growth and survival (Wu et al., 2023). Qn can enhance nutrient supply to distal parts of plant crowns (Scholz et al., 2007), and transport O_2_ to cells and nutrients to plants (Daley and Phillips, 2006). In addition, Qn represents a significant fraction of the total daily water loss (Zeppel et al., 2014), which influences the water balance for local and regional (Cirelli et al., 2016; Lombardozzi et al., 2017). Rn could maintain stomatal openness (de Dios et al., 2019) and promote transpiration and carbon fixation early at predawn (Fricke, 2019), supporting diurnal and seasonal transpiration in woody plants (Wang et al., 2012), especially in water-limited regions (Guo et al., 2023; Li et al., 2023). Moreover, Rn could prevent the hydraulic failure and drought-induced mortality during the dry season (Maherali et al., 2004; Mitchell et al., 2008).

Because of the different physiological significance of Qn and Rn on plant survival, studies have quantified and analyzed the influencing factors of these two components. According to the positive relationship between the VPD and En (Fuentes et al., 2013; Yu et al., 2018; Liu et al., 2023b), the relationship between VPD and En could determine the proportions of Qn and Rn. When VPD was low or close to zero, the values of En were almost the same as those of Rn. When En and VPD were strongly related, En was mainly used for Qn (Fisher et al., 2007; Phillips et al., 2010; Siddiq and Cao, 2018). Research has shown that the percentage of Rn to En can reach 40%-70% for *Quercus douglasii*, and the percentage of Qn to En is 30%-60% (Fisher et al., 2007). Because of the strong transpiration of the canopy during the day, the stem and leaf water potential decreased (Cavender-Bares et al., 2007; Kavanagh et al., 2007). Therefore, during the water potential gradient between the soil and plants, En was used for Rn to increase the leaf water potential and for water loss via cortex transpiration and cuticle transpiration (Chen et al., 2020). Before midnight, En was mainly used for Rn because of the difference between the root system and the soil of the tree after sunset; after midnight, En was used for Qn on semiaid and semihumid regions (Si et al., 2015; Su et al., 2022). Due to the controlling effect of leaf stomatal conductance on transpiration, Qn was deduced from the control of leaf stomatal conductance on whole transpiration during the day (Caird et al., 2007), while the stomata have different thresholds and sensitivities during the day and night (Buckley, 2005; Ogle et al., 2012).

Despite the growing body of literature documenting En and exploring its influencing factors, the ways in which the components of En vary with biophysical factors are still unclear. On the one hand, quantitative and dynamic characterization of these two processes is lacking. On the other hand, the relationships between En components and environmental factors are unclear. In the context of water shortage and climate change, studying the components of En is highly important to develop an in-depth understanding of the environmental response of plants and revealing the adaptability of plants, especially in water-limited regions. The objective of this study was to study the variation and influencing factors of the En components under contrasting soil moisture conditions, specifically, to (1) determine the amount and temporal dynamics of Qn and Rn at different time scales; (2) analyze the driving factors of Qn and Rn.

## 2 Materials and methods

### 2.1 Study site

**Figure 1:**
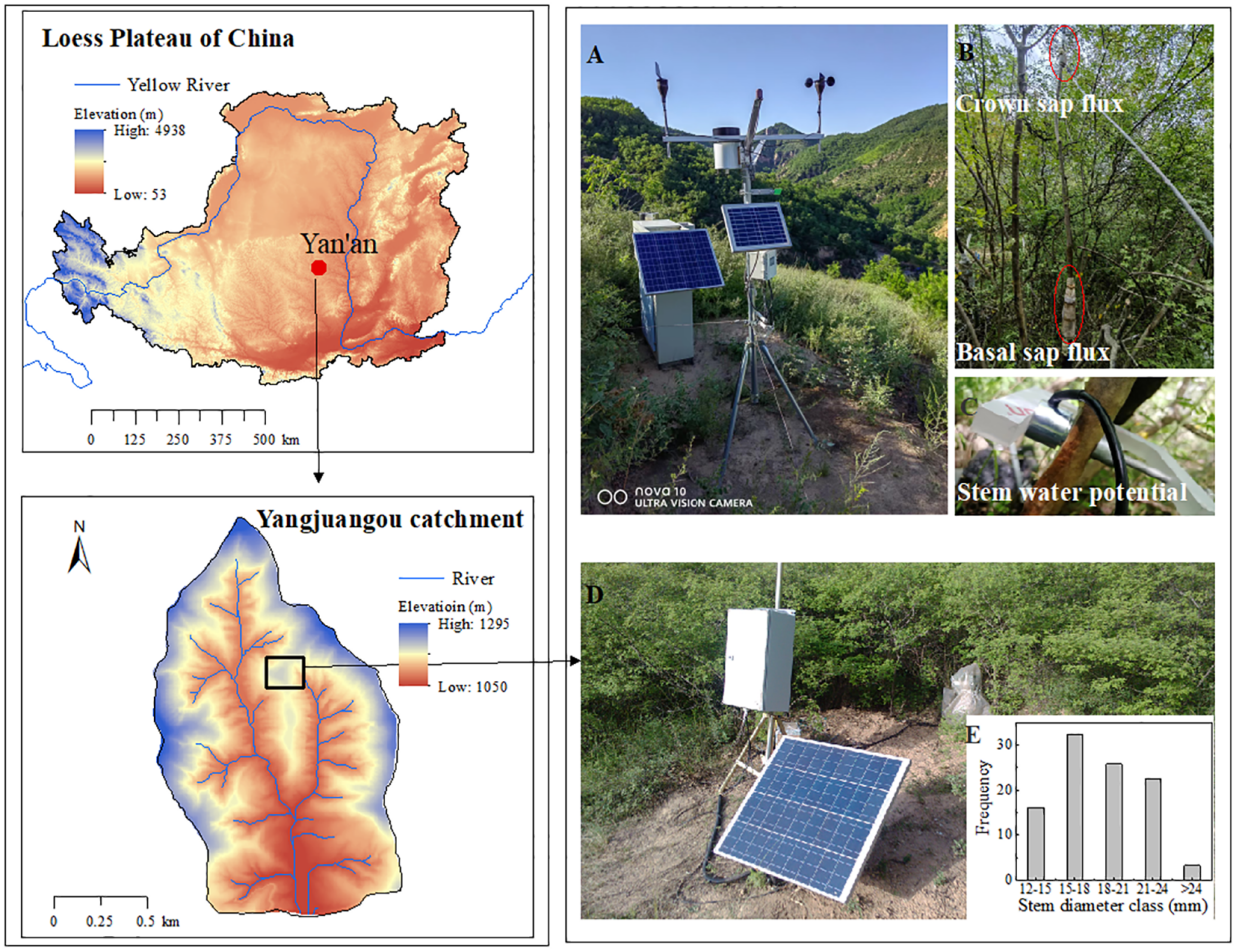
Location of the study area in the study plot of *V. negundo*. **(A)** The continuously recording meteorological station; **(B)** the position of sap flux density sensors; **(C)** the position of thermocouple stem psychrometer for stem water potential; **(D)** The sample plot of *V. negundo*; **(E)** the distribution of stem diameter of sample plot.

The study site was located in Yan’an City, Shaanxi Province, China, in the Yangjuangou catchment (36°42′N, 109°31′E, 1298 m in elevation) (**Figure 1**), where 531 mm of long-term mean annual rain occurred (from 1952-2012) and the mean temperature was 9.4 °C (Liu et al., 2023a). The original woody species were scarcely present because of intensive human activities. The site is dominated by the typical revegetated shrub species *Vitex negundo*, which is a perennial deciduous shrub that has been widely planted for ecological restoration since 1999 (Yuan et al., 2022), and the study plot experienced anthropogenic disturbances (**Figure 1D**). The height of *V. negundo* ranged from 1.4 to 2.2 m, with an average of 1.83 m. The sample plot was mainly composed of 31 scattered stems, and the stem diameter ranged from ranged from 12.54 to 25.61 mm, with average of 18.38 mm. The stems of diameter 15-24 mm, representing 80.65% of the total stems (**Figure 1E**), must have been responsible for the major part of the water use of *V. negundo.* The stems equipped with sap flow gauges were selected in this intermediated size range to represent of the most significant proportion of the stem population (**Table 1**).

**Table 1:** Profile of sample *V. negundo* shrubs used to conduct sap flux measurements.

Stem number	Stem diameter (mm)	Height (m)	Sapwood area (cm^2^)
1	18.00	1.74	2.54
2	19.21	1.82	2.90
3	22.34	1.85	3.92

### 2.2 Sap flow and stem water potential measurements

The En data of *V. negundo* individual stems were measured using the heat balance method because of its accuracy for shrub stems (Dugas et al., 1993; Hall et al., 1998). Different types of sensors were used according to the basal diameter of the stems (Dynamax Inc., Houston, TX, USA; Model SGA 13, and SGB 19). The theory and methodology of this method have been described in detail in previously study (Yue et al., 2008). Measurements were replicated using three individuals of *V. negundo*. The sap flow sensors were installed in each stem at the base (0.2 m above the ground) and at the top (the section of the canopy branch) of the live crown (**Figure 1B**). Data were sampled every 10 s, averaged and recorded at 30 min intervals. Measurements of sap flow density were made from June to September 2021 and 2022. Measurements began 2 days after the sensors were installed for data stability in June and continued until the end of September. Because the sap flux is correlated with tree size (Wang et al., 2012), daily sap flux of the sample stems was normalized with the stem area (g h^-1^ cm^-2^).

The stem water potential (Ψs, MPa) was automatically measured with thermocouple stem psychrometer (PSY, ICT International Pty., Australia). Thermocouple stem psychrometer could continuously record changes in plant water status, directly reflecting the energy needed by the plant to obtain water or the stress on the plant. According to the distribution of stem diameter in the sample plot, an ideal sample stem were selected with 18.42 mm diameter, representing the mean stem diameter of sample plot. The psychrometer was installed at 160 cm above the ground to measure the xylem water potential (**Figure 1C**). Before the instrument was installed, an installation section of 1cm wide and 5-6 cm long of stem was scraped with a knife, which need connect to the psychrometer. It was rinsed 3-5 times using deionized water and then rubbed with paper towels to ensure complete drying. A silicone grease seal was used for the connection between the psychrometer and stem. The gauge was protected using a shield wrapping of several layers of aluminum foil to reduce the effect of weather. The data were recorded at 15-min intervals in 2022.

### 2.3 Partitioning En into Qn and Rn

The quantities of daily and hourly En and Qn were the products of the nighttime sap flux density at the base and top at daily and hourly times, respectively. If the quantity of stored water in the crown was lower than that in the stem, the sap flux at the top was taken as Qn (Daley and Phillips, 2006), while the sap flux at the base represented En. The values of stem Rn were defined as the discrepancies between En and Qn. Negative Rn values indicated water withdrawal, while positive Rn values indicated excess water refilling depleted water stores. The sums of instantaneous changes in stored water were calculated for daytime and nighttime on hourly and daily time scales during the 2021 and 2022 from June to September.

### 2.4 Environmental factor measurements

An automatic weather station was installed in the open area of the catchment at approximately 100 meters away from the experimental field and 1.7 meters above the ground (**Figure 1A**). Air temperature (Ta, °C) and relatively humidity (RH, %) were measured using a HMP155 sensor (Vaisala, Finland). Solar radiation (Rs, W m^-2^) was monitored using a pyranometer (Model CNR 4, Kipp& Zonen B. V, the Netherlands). All climatic data were measured once per minute and recorded every 30 minutes from June 1 to September 30. Rainfall amount (mm) was recorded using a tipping-bucket rain gauge (TBRG) (Model RG3-M, Onset Computer Corporation, USA) mounted. The vapor pressure deficit (VPD, kPa) was calculated based on a Ta and RH equation (Campbell and Norman, 1998). Volumetric soil water content (SWC, %) at the study site was measured using EC-5 soil moisture probes (Decagon Devices Inc., Pullman, WA, USA) that were installed at seven depths below the soil surface (10, 20, 40, 80, 120, 160, and 200 cm). The environmental data were sampled and recorded at the same frequency as sap flow measurements.

### 2.5 Statistical analyses

To provide a conservative estimate of when stem refilling ceased, we used the time when radiation became <5 W m^-2^ or zero to define the beginning of the ‘night-time’ period in this study.

To identify the relationships between En components and environmental factors, between Rn and daytime sap flow and predawn Ψs, general linear regressions (y=a+b·x) were fitted to the data. Regression slopes were used as an indicator of overall sensitivity of Qn to the variation in biophysical variables. We applied one-way ANOVA to test the statistical significance of differences between Qn and Rn at a critical probability value of 0.05. Pearson’s correlation coefficient was used to estimate associated pairwise relationships between all variables. Stepwise multiple regression analysis was used to examine the contribution of Ta, RH and SWC in different soil layers to explaining the variations of Qn. All statistical analyses were performed with the SPSS software package (Version 19.0 for windows, SPSS Inc., USA). Linear fits were performed in Origin (Version 8.0, OriginLab Corp., USA).

In order to minimize the effect of rain events on nighttime sap flow measurements, only data collected during the non-rainy parts of the growing season were included in the analysis (Hayat et al., 2021).

## 3 Results

### 3.1 Environmental variables in the study area

**Figure 2:**
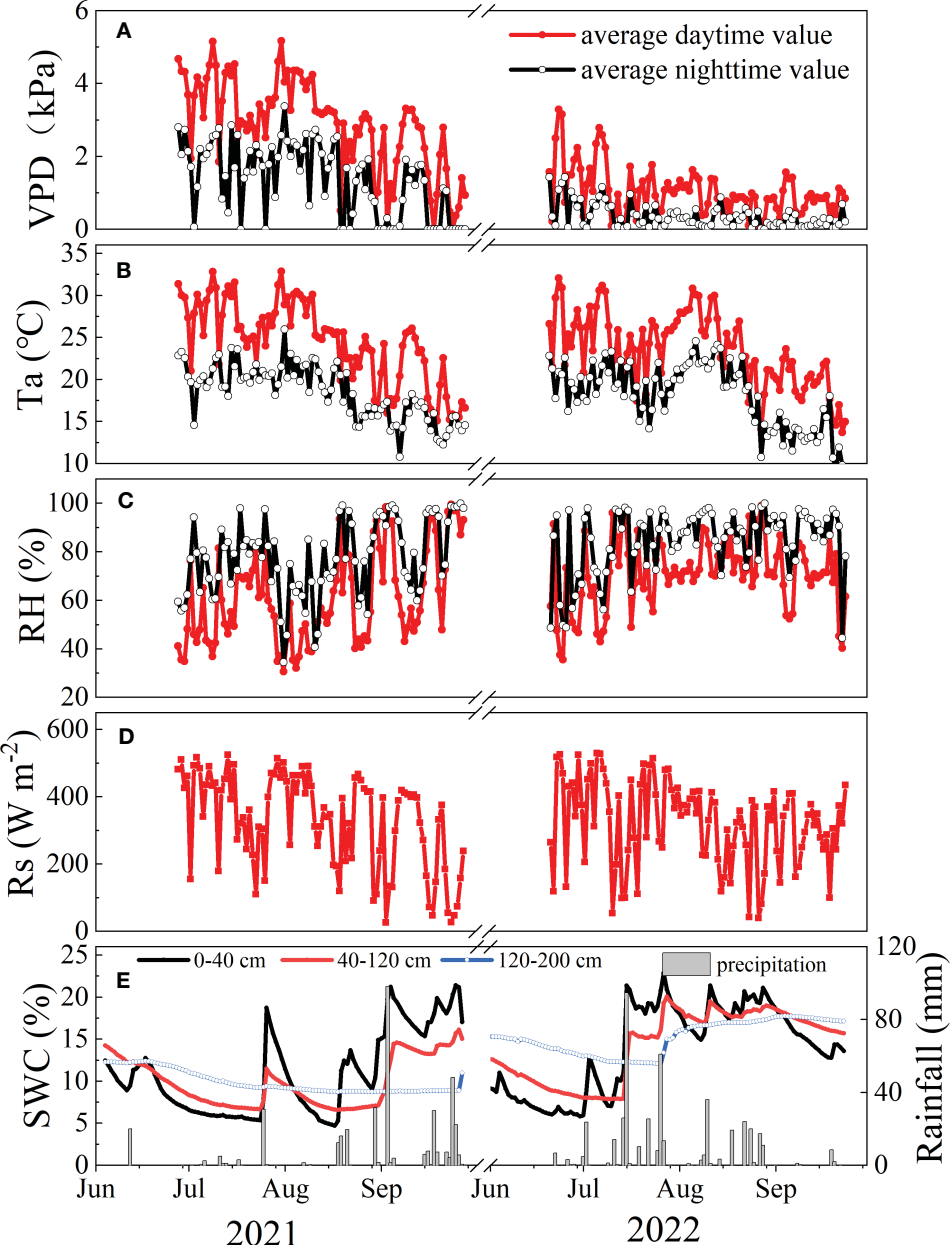
Temporal variations in **(A)** mean daily vapor pressure deficit (VPD, kPa), **(B)** mean air temperature (Ta, °C), **(C)** relatively humidity (RH, %), **(D)** daily solar radiation (Rs, W m-2) and **(E)** soil water content (SWC, %) at 0-40 cm, 40-120 cm, and 120-200 cm depth in the study area from June to September during 2021-2022.

The nighttime VPD was higher in 2021 than in 2022 (**Figure 2A**), while the opposite trend was observed for RH (*p*<0.01) (**Figure 2C**). Interannual variations in Ta and Rs exhibited no significant differences between the two years (*p*>0.05), with interannual means (± standard deviation) of 21.79 (± 0.29)°C, 202.68 (± 6.10) W m^−2^, respectively (**Figure 2B, D**). These hydrometeorological variables were lower at night than during the day (*p*<0.05). The total precipitation during the study period (June to September) was 371.40 mm in 2021 (a normal year), 450.87 mm in 2022 (a wetter year, 19.88% wetter than long-term average value (376.11 mm from 1952-2012)). Ta at night showed significant differences between the two years (*p*=0.03) (**Figure 2B**). In general, the SWC in the three soil layers (0-40 cm, 40-120 cm, 120-200 cm) in 2022 were significantly greater than those in 2021 (**Figure 2E**) (*p*<0.05).

### 3.2 Temporal variation of each En components

**Figure 3:**
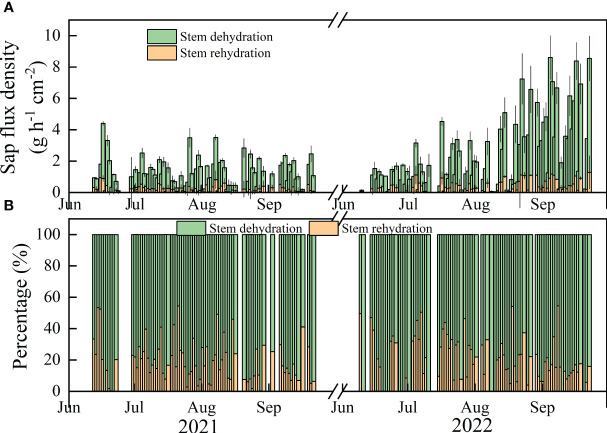
The temporal variation of Qn and Rn **(A)** and the values of Qn/En and Rn/En **(B)** during the study period. Black parts represent no values or the values were zero.

The values of Qn and Rn fluctuated during the growing season (**Figure 3**). The daily whole-stem Qn values during clear days ranged from 0 to 41.45 and 0 to 52.94 g cm^−2^ over the study period, with means of 4.68 (± 0.59) and 10.53 (± 1.31) g cm^-2^ per day in the main growing seasons in 2021 and 2022, respectively. The mean values of Rn were 0.86 (± 0.14) and 2.02 (± 0.27) g cm^-2^, ranging from 0 to 5.84 and 0 to 9.80 g cm^-2^ in 2021 and 2022, respectively.

**Figure 4:**
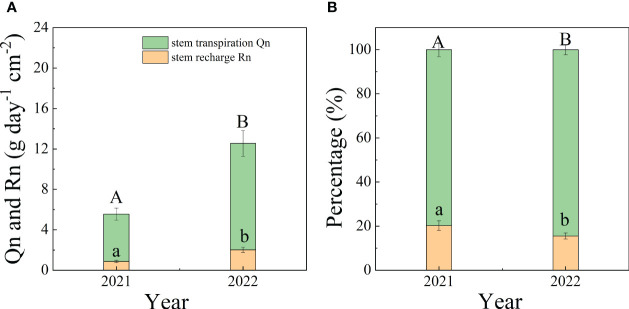
The interannual variation of Qn and Rn **(A)** and the mean percentage of Qn and Rn to En **(B)** during 2021-2022.

On monthly and interannual time scales, Qn was significantly greater than Rn (*p*<0.05). Qn and Rn were both significantly greater in 2022 than in 2021 (*p*<0.01), except for Qn in July. The maximum values of Qn and Rn were 15.89 g cm^-2^ day^-1^ in Sep 2022 and 2.55 g cm^-2^ day^-1^ in June 2022, respectively. The monthly mean values of Qn and Rn were 7.92 and 1.29 g day^-1^ cm^-2^ in 2021, 9.62 and 2.18 g day^-1^ cm^-2^ in 2022. The ratio of Qn/En was lower in 2021 (79.76%) than in 2022 (83.91%) (*p*=0.03). The average values of Qn/En and Rn/En in in two years were 81.83% and 18.17%, respectively (**Figure 4**).

### 3.3 Diurnal variation in Rn and Ψs

**Figure 5:**
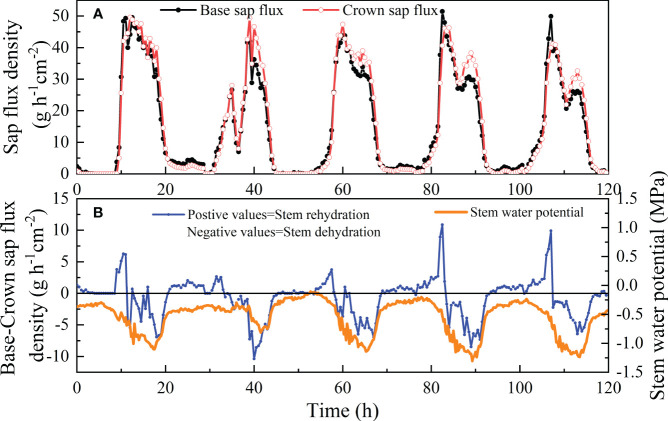
Diurnal course of crown and basal sap flux **(A)** and the difference between basal and crown sap flux and stem water potential **(B)** in a sample shrub of *V. negundo* from July 4 to 8 in 2022. Black lines represent the sap flux at the base of live crown and grey lines represent sap flux at 160 cm height. The water storage flux is calculated as the difference between sap flux at the base of the live crown and sap flux at 160 cm height.

The time series for five days from July 4 to 8 in 2022 were chosen to provide typical basal and crown En and Ψs patterns on clear sunny days. During the morning hours, the water balance of the shrub stem between the base and top was positive, the inflow from the lowermost stem was lower than the outflow to the upper stem (**Figure 5**), and Ψs was relatively high at predawn. The balance became negative when the input into the stem was lower than the output into the stem until approximately after 9 p.m. with decreasing Ψs. Plant depleted water storage was recharged at night until the early morning hours of the next day transpiration resumed. En returned to almost zero before sunrise. The Ψs decreased from sunrise until the afternoon at approximately 6 p.m., with a minimum of -1.24 MPa (**Figure 5B**). Then, Ψs increased until predawn, corresponding to the recharge period of the stem.

### 3.4 The influencing factors on sap flow components

#### 3.4.1 Environmental controls on Qn and Rn

**Figure 6:**
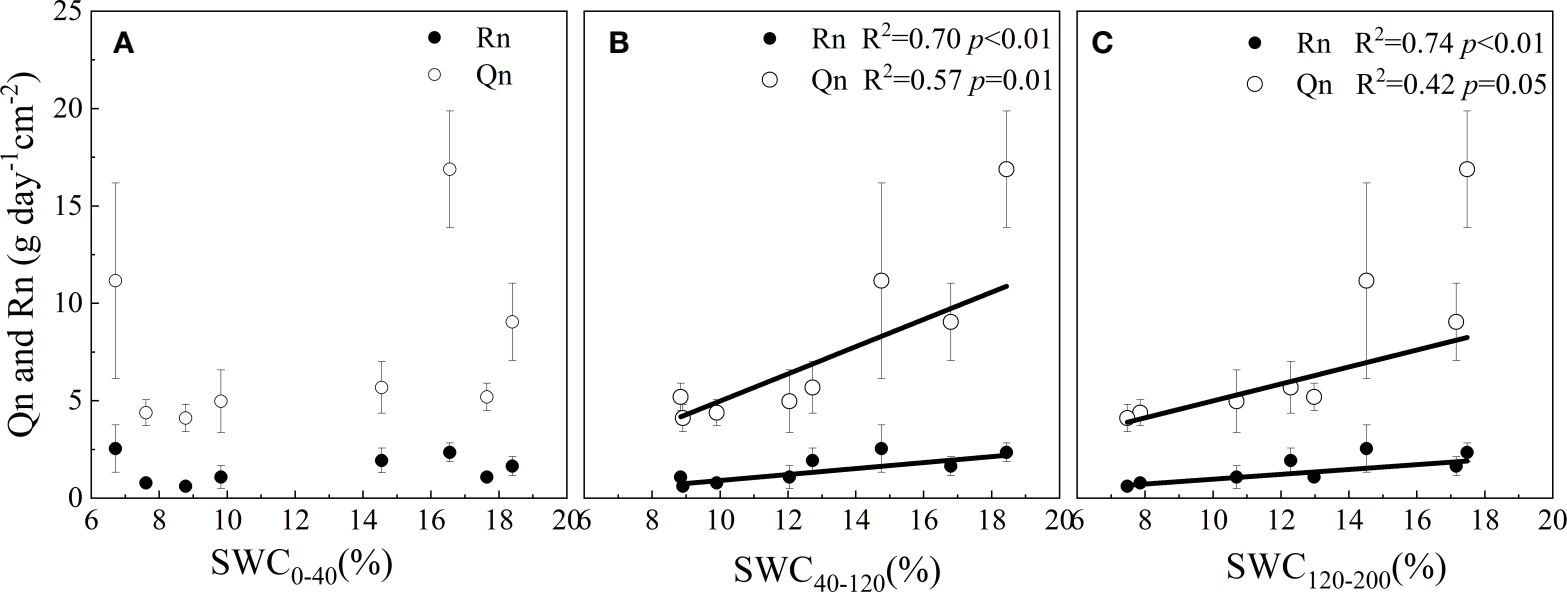
Sensitivity of Qn and Rn to soil moisture in different soil layers (0-40 cm **(A)**, 40-120 cm **(B)** and 120-200 cm **(C)**).

The correlation coefficient (R) of the Qn and environmental factors differed on different time scales. On an hourly time scale, Qn and Rn were affected by Ta, VPD, RH and SWC in different layers. On a daily time scale, Qn was negatively correlated with RH and SWC_0-120 cm_ in 2021 and with VPD, Ta and SWC in 2022. Qn increased with increasing SWC, whereas Rn was not affected by meteorological or SWC factors (**Table 2**). On the monthly time scale, Qn and Rn were affected by the SWC in the 40-200 cm layer, not by meteorological factors (**Figure 6**).

**Table 2:** Correlations between daily Qn and Rn of *V. negundo* and air temperature (Ta), relatively humidity (RH), vapor pressure deficit (VPD), soil water content at different layers in 2021 and 2022.

Year			Qn	Rn	Ta	RH	VPD	SWC_0-40cm_	SWC_40-120cm_	SWC_120-200cm_
2021	Hourly	Qn	––	0.39^**^	0.28^**^	-0.46^**^	0.41^**^	0.01	0.14^**^	0.20^**^
		Rn	0.39^**^	––	0.13^**^	-0.20^**^	0.20^**^	-0.04	0.10^*^	0.16^*^
	Daily	Qn	––	0.54^**^	-0.14	-0.44^**^	0.20	0.40^**^	0.34*	0.23
		Rn	0.54^**^		0.17	-0.20	0.22	-0.07	0.01	0.23
2022	Hourly	Qn		0.73^**^	-0.02	-0.35^**^	0.25^**^	0.12^**^	0.21^**^	0.29^**^
		Rn	0.73	––	0.03	-0.24^**^	0.17^**^	0.16^**^	0.20^**^	0.21^**^
	Daily	Qn	––	0.70^**^	0.45^**^	0.19	0.30^*^	0.29^*^	0.43^*^	0.51^*^
		Rn	0.70^**^	––	0.10	0.16	0.06	0.16	0.17	0.26

** Significant at p<0.01, *significant at p<0.05.

Environmental factors did not adequately explain the amount of Qn. The factors RH and SWC explained 37% of the variation in Qn in 2021. The SWC alone explained 27% of the variation in Qn in 2022. The explanation of the SWC was more significant in wetter years (**Table 3**).

**Table 3:** Regression analysis models of nocturnal transpiration (Qn) versus air temperature (Ta), relatively humidity (RH) and soil water content (SWC) in different soil layers.

Year	Equation	Adjusted R^2^	Explanation degree	
			Meteorological	SWC
2021	Qn =2.281-0.025RH+0.054SWC_0-40cm_	0.37	19%	18%
2022	Qn =-4.89 + 0.48SWC_120-200cm_	0.27	0	27%

#### 3.4.2 The mutual effect of Qn and Rn

**Figure 7:**
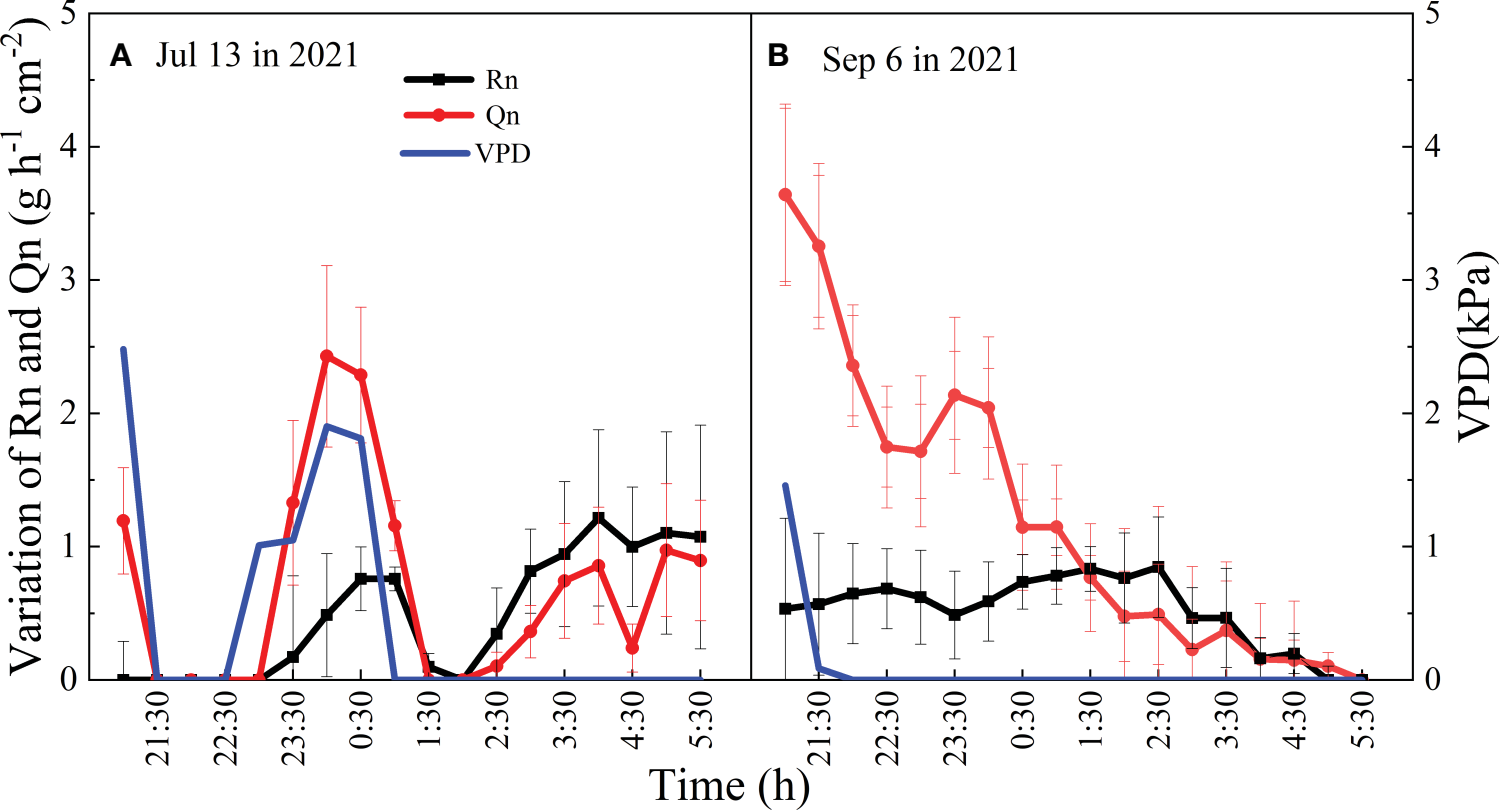
Mean values and temporal variation of Qn and Rn during the night of Jul 13 **(A)** and Sep 6 **(B)** in 2021.

Daily Qn and Rn also influenced with each other, and the correlation coefficient increased with increasing precipitation (R=0.54 in 2021, R=0.70 in 2022) (**Table 2**). Qn and Rn also exhibited synergistic relationships. When the night was rainless, Qn and Rn both showed a decreasing trend, which was similar to the change in VPD, and Qn was greater than Rn (*p*<0.05). When VPD and wind speed were nearly zero, Qn increased with the increasing Rn, and Rn was greater than Qn (**Figure 7**).

## 4 Discussion

### 4.1 The magnitude and occurrence of Qn and Rn

In our experiment, the mean hourly Qn and Rn values were 1.65 and 0.31 g cm^-2^, respectively, and the two fluxes were both greater in the wet year. The mean percentage of Qn to En in this study was 81.83%. Consequently, Qn was the predominant contributor to En during the study period. Although the percentages of En to daytime sap flow were greater in water-limited regions (Snyder et al., 2003; Wang et al., 2012), the proportions of Qn and Rn to En did not reach the same conclusion in previous studies. Some studies have shown that En is mainly used for refilling water in trunks. Researchers have shown that the percentage of Rn to En was 40%-70% for *Quercus douglasii* (Fisher et al., 2007), ranging from 14.7 to 30.3% for *Acacia mangium* (Wang et al., 2012), and approximately 90% for *Acer truncatum* (Wu et al., 2020), indicating a greater allocation of En to Rn. In temperate woodlands, Qn/En was 50-70% for two co-occurring evergreen species (*Eucalyptus parramattensis* and *Angophora bakeri*) (Zeppel et al., 2010). Studies showed that Rn was strongly affected by tree features, with the exception of environmental factors, such as plant size (Horna et al., 2011); plant sapwood (Carrasco et al., 2015); basal area (Wang et al., 2012) and plant canopy (Williams et al., 2021), which exhibited greater capacitance. Because of the significance of Rn for daytime water loss, the amount of water stored is greater for trees than for shrubs. The volume of leaves and small branches above the top sensor were not considered, which may lead to underestimation of stem refilling volume in this study.

Studies have observed that stem refilling for *Eucalyptus saligna* finishes by 23:00 h because daytime water use causes a deficit in the internal water storage of plants (Daley and Phillips, 2006; Zeppel et al., 2011), which can be recharged through nighttime stem refilling for tree species. However, in the present study, the refilling of capacitors increased from dusk to predawn, while Ψs stopped increasing until predawn, suggesting that the nocturnal recharge of stem water storage was essentially complete during the night (**Figure 5**). The correlation between Qn and Rn and its variations suggested that water recharge and transpiration were synchronous at night (**Table 2**, **Figure 7**). Research has also shown that there is no discernible distinction between Qn and Rn during the process of En (Daley and Phillips, 2006; Huang et al., 2017). Therefore, using only the time to distinguish Qn from Rn would introduce error.

### 4.2 The affecting factors of Qn and Rn

#### 4.2.1 Meteorological and SWC factors

Our results showed that the Qn of *V. negundo* was influenced by the combination of meteorological and SWC factors. The variance in Qn in the model explained by RH and SWC was 37%, which was relatively greater than that explained by RH alone. The response of Rn or En to VPD was observed in many studies (Chen et al., 2020; Wu et al., 2020), because a high VPD affected the differences in water potential between the leaves and atmosphere, leading to the occurrence of Qn (Kavanagh et al., 2007). The sensitivity of En to VPD was also not significant for *A. mangium* (Wang et al., 2012). The reason may be that the effect of SWC overwrites that of VPD on daily time scale. The initial variation patterns of Qn and Rn were similar to the variation in VPD during the first 3-4 h after dusk (**Figure 7A**), indicating that the driving factors of Qn were meteorological factors at the hourly time scale. Qn and Rn occurred simultaneously (Daley and Phillips, 2006) and the positive correlation between Qn and Rn suggested that Qn may drive the Rn processes. Additionally, the correlation between Qn and Rn was more significant in relatively wetter years (**Table 2**). This suggested that higher En in 2022 increased Qn, which drove increased Rn, and Rn was greater in 2022.

Because the effects of SWC on Qn and Rn differed, the response of En to SWC was not conclusive (Zeppel et al., 2010; Chen et al., 2020). The contributions of Rn to En were 20.24% in 2021 and 16.09% in 2022, which were greater in the normal year than in the wet year. Notably, the ratios of En to daytime sap flow were greater in water-limited regions, such as the western United States (10-32%) (Snyder et al., 2003), northwestern Australia (Pfautsch et al., 2011) and a Mediterranean holm oak forest (Barbeta et al., 2012). A study showed that the En of paper birch was mostly used for Qn under adequate SWC conditions (Daley and Phillips, 2006). The effect of SWC on Qn was high in wet year (**Table 3**), so Qn increased when the rainfall amount increased. The value of Rn was not influenced by meteorological factors in either wet or dry year. The occurrence of Qn and Rn was synchronous, so Rn simultaneously increased with Qn and was correlated with SWC in wet years. Under dry conditions, the opening of stomatal conductance decreased, which weakened the penetration ability of water to plant leaves and reduced Qn (Donovan et al., 2003; Ludwig et al., 2006). This is consistent with the greater allocation of En to Qn, decreasing the value of Rn/En in wet years.

#### 4.2.2 Endogenous regulation

The influence of SWC on Rn did not adequately explain the variation in Qn on the daily time scale in this study. When VPD was nearly zero, an increase in Rn initially occurred, followed by an increase in Qn (**Figure 7B**). We suggest that the effect of circadian dynamics overwrites the effect of VPD under certain circumstances. Studies have shown that the endogenous regulation of stomatal conductance, regarded as circadian-driven regulation, affects the water use of whole trees at night (Caird et al., 2007; Easlon and Richards, 2009). The variation in Rn in this study indicated that the endogenous regulation also drove the variation in Rn.

The occurrences of Qn and Rn at an hourly time scale were synchronous regardless of the major or minor effect of meteorological factors. When the effect of meteorological conditions on Qn was minor, Rn was greater than Qn, and Rn drove Qn by circadian clock; moreover, the effect and explanation of endogenous regulation on Rn variation was not conclusive. de Dios et al. (2013) reported that stomatal opening occurs 3-12 h after dusk with decreasing VPD because of physiological mechanisms. The percentage of the circadian clock explained by En was 23%-56% in controlled experiment (de Dios et al., 2013). We propose further studies to examine the combined effect of environmental factors and endogenous regulation on whole-tree water use under natural conditions.

### 4.3 The significance of Qn and Rn

Variation in the components of En may be an adaptation in response to resource deficiencies and may provide eco-physiological advantages for plant growth. In this study, En is mainly used for Qn. Thus, in species like *V. negundo*, increased evaporation demand during the night may affect the plant water use and ecological water balance of plant ecosystem (Lombardozzi et al., 2017). Studies also proposed the Qn may improve oxygen supply to the sap wood or prevent of CO_2_ build-up in leaves during the night (Marks and Lechowicz, 2007), reduce leaf surface temperature (Peraudeau et al., 2015). It may also transport nutrients to the roots and distal parts of the plant, which is important in nutrient-limited but water-sufficient areas (Scholz et al., 2007). In water-limited areas, Qn may reduce the water potential of plant leaves and inhibit hydraulic redistribution.

**Figure 8:**
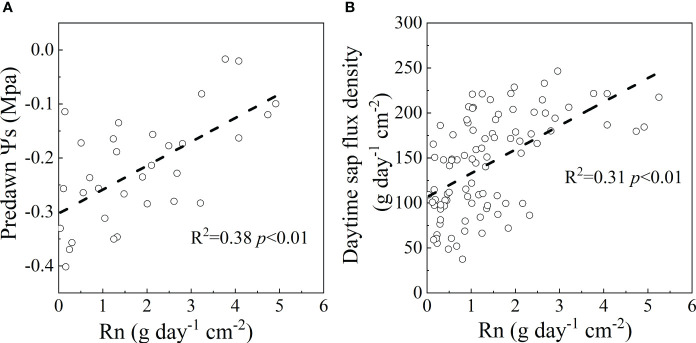
The response of predawn Ψs in 2022 **(A)** and daytime sap flow in 2021 and 2022 **(B)** to nighttime water recharge in rainless days.

Although Rn was a small fraction of En in this study, the predawn Ψs in 2022 and the daytime sap flow in both years were positively correlated with Rn (R^2^ = 0.38, *p*<0.01 for predawn Ψs; R^2 ^= 0.31, *p*<0.01 for daytime sap flow) (**Figure 8**), which may facilitate stomatal opening and subsequent carbon fixation during the early morning. Our results also showed that En was correlated with daytime sap flux (**Figure 8B**) (Wang et al., 2012), indicating that an amount of nighttime sap flux was used for stem water recharge as a result of high water loss during the day (Huang et al., 2017). The mean Rn contributed 18.17% of the En in this study suggesting the importance of Rn for the adaptive strategy of shrubs to soil water scarcity. The contribution of water storage in the stem to transpiration accounted for 10-20% of the daily transpiration for Japanese red pine and an oak forest (Kobayashi and Tanaka, 2001). Therefore, an estimation of Rn could provide an in-depth understanding of plant adaptation to drought stress. If the VPD_night_ increased with the warmer and drier condition under climate change, the percentage of Rn will continue to increase with Qn, which would change the adaptability to the environment. Therefore, the occurrence and amount of Rn are more important for plant survival, especially in water-limited regions.

### 4.4 Uncertainties

There are some limitations in this study, including the low sample replication and systematic errors of gauge heater. The replication rate of this study for the sample size may not represent all the morphological and physiological features of the species and there is a possibility of overlooking some specific stem sap flow information. Due to field conditions and equipment limitations, 3-6 samples have been commonly used in previous studies for monitoring plant sap flow or transpiration, such as three samples (Yu et al., 2016, 2018), four samples (Wu et al., 2020) and six samples (Yue et al., 2008). On the other hand, the presence of measurement instrument errors would be another limitation in this study. Although the results may be acceptable using this method (Flo et al., 2019), the systematic errors in the heat balance method were not considered in this study, such as radial variations in sap flow, species specific differences in the parameters setting in the instrument (Moro et al., 2004; Repo et al., 2008) and the environmental conditions that cause temperature differences along the stem (Shackel et al., 1992). These uncertainties need to be improved in future studies.

## 5 Conclusions

The mean contribution of Qn to En was 79.76% and 83.91% in 2021 and 2022, respectively, indicating that the nighttime sap flux was mostly used for transpiration. The values of Qn and Rn were greater in wet year than that in normal year, while the Rn/En was greater in normal year than in wet year. The main controlling factors of Qn were environmental factors (RH and Ta) on a daily time scale, and SWC on monthly time scale. At an hourly time scale, Qn increased with the increasing VPD, and Qn drove the variation in Rn. However, Rn was greater than Qn when VPD was nearly zero, and Rn drove the occurrence of Qn when the effect of the environment on Qn was minor. The correlation between Qn and Rn suggested the two fluxes were synchronous in both normal and wetter year. Rn was positively correlated with daytime sap flux and predawn Ψs, indicating the ecophysiological significance of Rn for plant survival, especially in water-limited regions. Additional research is necessary to fully understand the effect of endogenous regulation at night. Furthermore, the uncertainty caused by nighttime water recharge on canopies deserves further investigation.

## Data availability statement

The raw data supporting the conclusions of this article will be made available by the authors, without undue reservation.

## Author contributions

WF: Conceptualization, Data curation, Formal analysis, Funding acquisition, Investigation, Methodology, Writing – original draft, Writing – review & editing. JL: Conceptualization, Writing – review & editing. NL: Funding acquisition, Investigation, Supervision, Writing – review & editing. RL: Data curation, Writing – review & editing.
